# On-treatment modified Glasgow Prognostic Score (mGPS) in hepatocellular carcinoma treated with atezolizumab and bevacizumab provides prognostic information

**DOI:** 10.3389/fimmu.2025.1599143

**Published:** 2025-10-09

**Authors:** Tessa Hattenhauer, Rebekka Mispelbaum, Annkristin Heine, Katjana Schwab, Peter Brossart, Niklas Klümper, Jonas Saal

**Affiliations:** ^1^ Medical Clinic III for Oncology, Hematology, Immune-Oncology and Rheumatology, University Hospital Bonn (UKB), Bonn, Germany; ^2^ Center for Integrated Oncology Aachen/Bonn/Cologne/Düsseldorf (CIO-ABCD), Bonn, Germany; ^3^ Department of Urology, University Hospital Bonn (UKB), Bonn, Germany; ^4^ Institute of Experimental Oncology, University Hospital Bonn (UKB), Bonn, Germany

**Keywords:** HCC, biomarkers, immunotherapy, checkpoint-inhibition, mGPS

## Abstract

**Background & aims:**

Hepatocellular carcinoma (HCC) is associated with high cancer-specific mortality. While immune-checkpoint inhibitors (ICIs) improved overall survival (OS) compared to tyrosine kinase inhibitors, biomarkers predicting response to ICI in HCC are lacking. This study investigates the prognostic value of serum-based prognostic scores in patients with HCC receiving atezolizumab and bevacizumab.

**Methods:**

This *post-hoc* study analysis evaluates data from the phase 3 IMbrave150 trial, comparing atezolizumab plus bevacizumab to sorafenib in patients with unresectable HCC. 212 patients were included in the analysis. The prognostic value of imaging was compared to albumin, C-reactive protein (CRP) and interleukin-6 (IL-6), as well as composite scores, including the modified Glasgow Prognostic Score (mGPS) after three cycles of therapy. For further analysis, patients were classified in three risk groups according to each scoring system (low-risk, intermediate-risk and high-risk).

**Results:**

The on-treatment mGPS, assessed 9 weeks post-treatment initiation, predicted OS with hazard ratios of 2.31 (95% CI 1.39–3.83, p < 0.001) for intermediate-risk and 3.40 (95% CI 3.07–5.59, p < 0.001) for high-risk, compared to low-risk groups, showing greater accuracy than RECIST imaging. Albumin, CRP, and IL-6 were individually good prognostic indicators, with albumin/CRP (ACR) and albumin/IL-6 (AIR) ratios having the highest prognostic power (c-index: ACR 0.66 (95% CI 0.61–0.71), AIR 0.67 (0.62–0.72), mGPS 0.62 (0.57–0.66)). Multivariable analysis confirmed serum-based scores’ prognostic value independent of imaging. Serum-based scores significantly correlated with survival in patients with stable disease (SD, 79% of patients) or progressive disease (PD, 12% of patients).

**Conclusions:**

The on-treatment mGPS, as well as ACR and AIR, predicted outcomes in patients with HCC independent of and more accurately than radiological staging. Since, the mGPS is the most cost effective and widely validated score, we consider it best suited for clinical use. Prospective validation is needed to confirm these findings.

## Introduction

Liver cancer is the sixth most diagnosed cancer. The predominant subtype is hepatocellular carcinoma (HCC) (1). Despite increasing therapy options, HCC is still associated with a high cancer specific mortality. For many years, tyrosine kinase inhibitors (TKIs) have been the most effective treatment option for advanced HCC (2). In recent years, immune-checkpoint inhibitors (ICIs) have emerged as a new promising treatment approach. Checkpoint molecules such as programmed cell death protein 1 (PD-1) and programmed death ligand 1 (PD-L1) physiologically regulate duration and intensity of immune responses. Tumor cells exploit this control mechanism and create an immunosuppressive tumor microenvironment favoring tumor growth. By blocking inhibitory checkpoint signaling pathways, immunotherapy induces tumor-directed CD8^+^ T cells, playing a key role for anti-tumor immunity (3). Clinical trials evaluating ICIs in HCC have shown improved overall survival (OS) compared to TKI treatment (4, 5).

However, not all patients profit from ICI treatment and reliable biomarkers for therapy response are missing in patients with HCC (6). While predictive biomarkers guide the selection of the initial treatment, on-treatment biomarkers help to decide between continuation and switch to an alternative therapy regime during the course of treatment. PD-L1 status, being an established biomarker for other cancer types, has limited predictive value in HCC (7). In clinical practice, treatment response to immunotherapy is currently assessed using imaging evaluated by RECIST criteria (8). Especially in the era of immuno-oncology, imaging has various limitations, particularly in patients with stable disease (SD). This subgroup is characterized by a high heterogeneity in progression-free survival (PFS)and OS. There is unmet clinical need for new biomarkers allowing real-time assessment of treatment response and outcomes in addition to imaging techniques (9).

Previous studies indicate a prognostic value of baseline Albumin and C-reactive protein (CRP) levels for treatment outcome in HCC patients (10, 11). In line with this, the inflammation-based “modified Glasgow Prognostic Score” (mGPS), combining both markers, is an independent prognostic factor in HCC (12).

In lung, renal and urothelial cancer, on-treatment mGPS was highly prognostic in patients with SD in the first staging (9, 13, 14). To date, it has not been investigated whether re-assessing the mGPS during treatment can predict treatment outcome and overcome the limitations of imaging-based therapy monitoring in HCC. We explore the role of on-treatment mGPS compared to similar serum-based biomarkers for prognostication in patients with HCC receiving atezolizumab and bevacizumab in the pivotal phase III trial IMbrave150 (4).

## Methods

The here presented data are extracted from the *post hoc* analysis of the IMbrave150 trial. This randomized phase 3 trial investigated atezolizumab and bevacizumab in comparison to sorafenib in patients with unresectable HCC (4). Patients were eligible if they were 18 years or older and had locally advanced, metastatic, or unresectable HCC that could be assessed according to RECIST version 1.1 criteria. Additional inclusion criteria were an Eastern Cooperative Oncology Group (ECOG) performance status of 0 or 1, Child-Pugh class A liver function, and adequate hematologic and organ function. Patients were excluded if they had received prior systemic therapy for HCC, had a history of autoimmune disease, were co-infected with hepatitis B or C virus, or had untreated or incompletely treated esophageal or gastric varices associated with a high risk of bleeding.

The intervention arm received 1200 mg of atezolizumab plus 15 mg/kg of bevacizumab administered intravenously every 3 weeks. Treatment was continued until the occurrence of unacceptable toxicity or loss of clinical benefit, with the option to continue beyond disease progression if clinically justified. The co-primary endpoints were OS and PFS (4).

Data was available for a total of 279 patients treated with atezolizumab and bevacizumab. Imaging results from the first staging, albumin and CRP values were available for 212 patients (**Supplementary Figure S1**). Serum Interleukin-6 (IL-6) concentration was reported for 201 patients. The on-treatment mGPS was available for 212 patients at the time of initial radiologic staging (with a median of 64 days for mGPS assessment and a median of 41 days for imaging).

Therapy response to ICIs was classified in accordance with the RECIST version 1.1. Patients were grouped based on the staging results in complete response (CR), partial response (PR), SD or progressive disease (PD). The modified Glasgow Prognostic Score (mGPS) was calculated by assigning 1 point for an elevated CRP concentration (>10 mg/L) and, if CRP levels are elevated, an additional point for decreased serum albumin (<35 g/L). Patients are categorized into low risk (0 points), intermediate risk (1 point), and high risk (2 points) groups based on their mGPS scores. The albumin/CRP ratio (ACR) and albumin/IL-6 ratio (AIR) were calculated by dividing the serum concentration of albumin (in g/L) by the concentration of CRP (in mg/L) or IL-6 (in ng/L), respectively. Patients were classified in low risk (first tercile), intermediate risk (second tercile), and high risk (third tercile).

Imaging results and mGPS risk groups were evaluated for their correlation with OS. Additionally, individual biomarkers including albumin, CRP, and IL-6, as well as ACR and AIR, were analyzed in relation to OS. In patients with PR, SD or PD, OS was further examined in correlation with mGPS, ACR, and AIR to assess their prognostic value within these subgroups.

### Statistical analysis

Investigator-assessed OS was utilized for survival analyses. Outcomes were evaluated using univariate Kaplan-Meier estimation and tested with the log-rank test as well as using the Cox regression model. Score performance was evaluated using the concordance index (c-index) for Cox regression models. All analyses were carried out in R studio, version 1.4 (R Foundation for Statistical Computing) using the packages “survival”, “survminer”, “ggplot2” and “gtsummary”, within the vivli.org secure research environment, a remote desktop tool. According to Vivli guidelines, only research results, not patient-level data, are permitted to be exported from the environment. Consequently, we do not possess copies of the patient-level datasets. A two-sided p-value less than 0.05 was deemed statistically significant for all statistical tests. None of the analyses was pre-specified in the original trial protocol.

The data were provided by vivli.org. Approval for our study was granted by Vivli’s independent review panel, which includes an ethics branch. We adhered to the Transparent Reporting of a Multivariable Prediction Model for Individual Prognosis or Diagnosis (TRIPOD) reporting guideline throughout this study.

## Results

A total of 212 patients treated with atezolizumab and bevacizumab in the IMbrave150 trial were included in further analysis. The mean age was 64 years (range: 56–71 years). 82% of patients were men and 18% women. 62% of patients had a ECOG performance status of 0 and 38% had an ECOG performance status of 1 before the start of ICI treatment. The reported Child-Pugh classification of 72% patients was A5 and of 28% patients A6. Baseline characteristics of the patients are shown in **Supplementary Table S1**.

**Figure 1:**
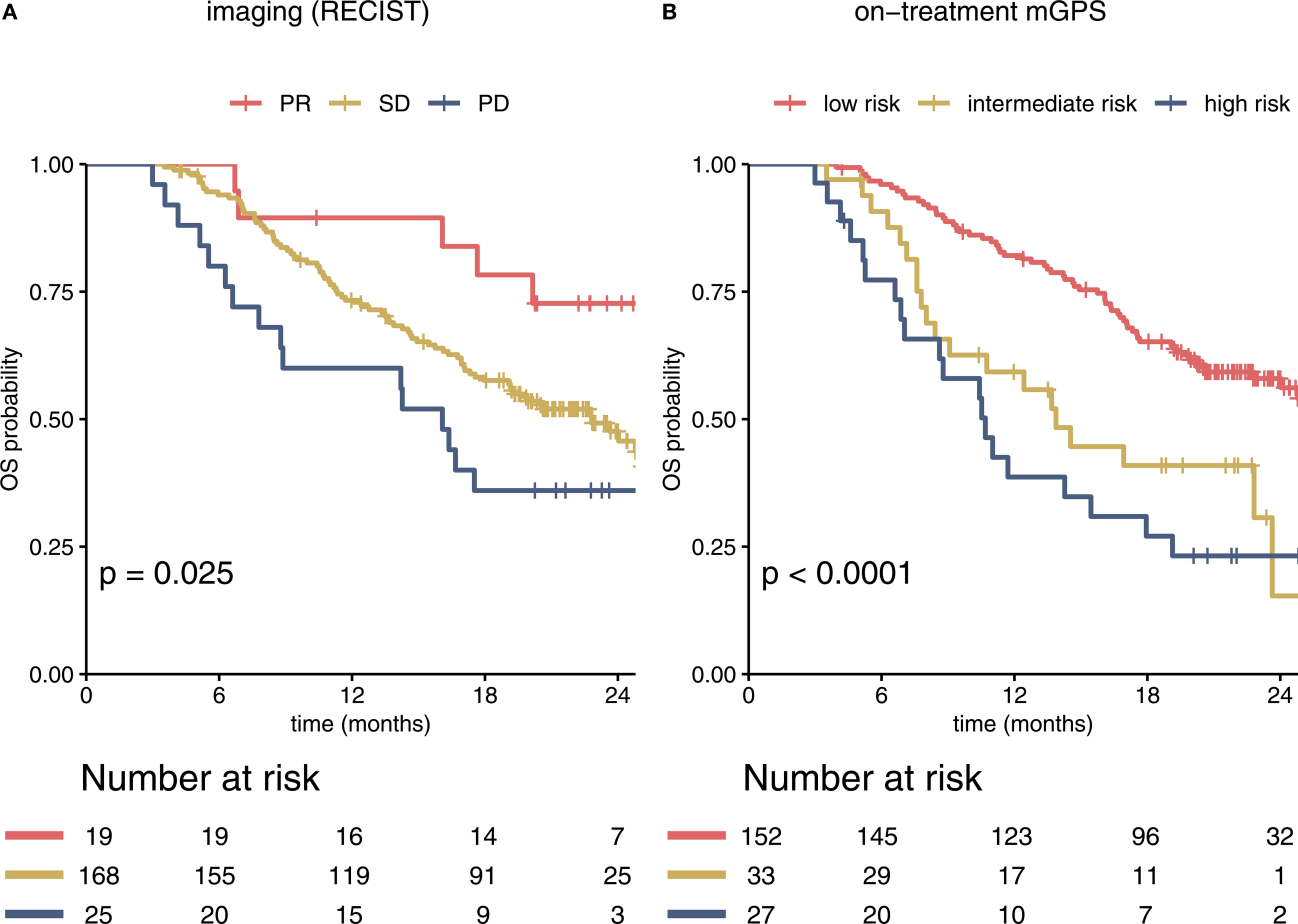
Imaging and the on-treatment mGPS predict survival in immunotherapy-treated patients in the IMbrave150 trial. Kaplan-Meier curves for RECIST based imaging and on-treatment mGPS are shown **(A)**. PR, SD and PD in the first staging correlated significantly with estimated OS. Most patients were classified as SD. **(B)** In comparison to imaging based stratification, risk groups according to mGPS at cycle 4 showed stronger correlation with OS within the overall cohort (c-index 0.62 (95% CI 0.57 – 0.66) for mGPS vs 0.56 (0.52 – 0.60) for imaging). mGPS, modified Glasgow Prognostic Score; PR, partial response; SD, stable disease; OS, overall survival; PD, progressive disease; OS, overall survival.

Therapy response was evaluated by CT scan after 2 cycles (i.e., 6 weeks after the start of treatment) with a median time of 41 days after the first ICI application. An initial objective response was detected in 19 of 212 patients (8.96%), all of whom showed a PR. 168 (79.25%) patients were classified as SD and 25 (11.79%) patients showed primary PD in the first staging. As expected, response assessed according to RECIST predicted survival, with 12-month OS rates of 89% (95% CI 77 – 100%) for responders, 73% (67 – 80%) for patients with SD and 60% (44 – 83%) for patients with PD. The hazard ratio (HR) for death was 2.41 (95% CI 0.98 – 5.94, p = 0.057) for patients with SD and 3.76 (1.38 – 10.3, p = 0.01) for patients with PD compared to patients with PR (**Figure 1A**).

To assess the prognostic value of the on-treatment mGPS, serum CRP and albumin levels were analyzed. As CRP values were not available for the 6-week timepoint (timepoint of first staging), we calculated the mGPS at the beginning of cycle 4 (median 64 days after the start of treatment, **Figure 1B**). The on-treatment mGPS predicted outcomes with high accuracy, outperforming RECIST based imaging in predicting the risk of death [c-index for imaging: 0.56 (95% CI 0.52 – 0.60), for on-treatment mGPS at cycle 4: 0.62 (0.57 – 0.66)].

**Figure 2:**
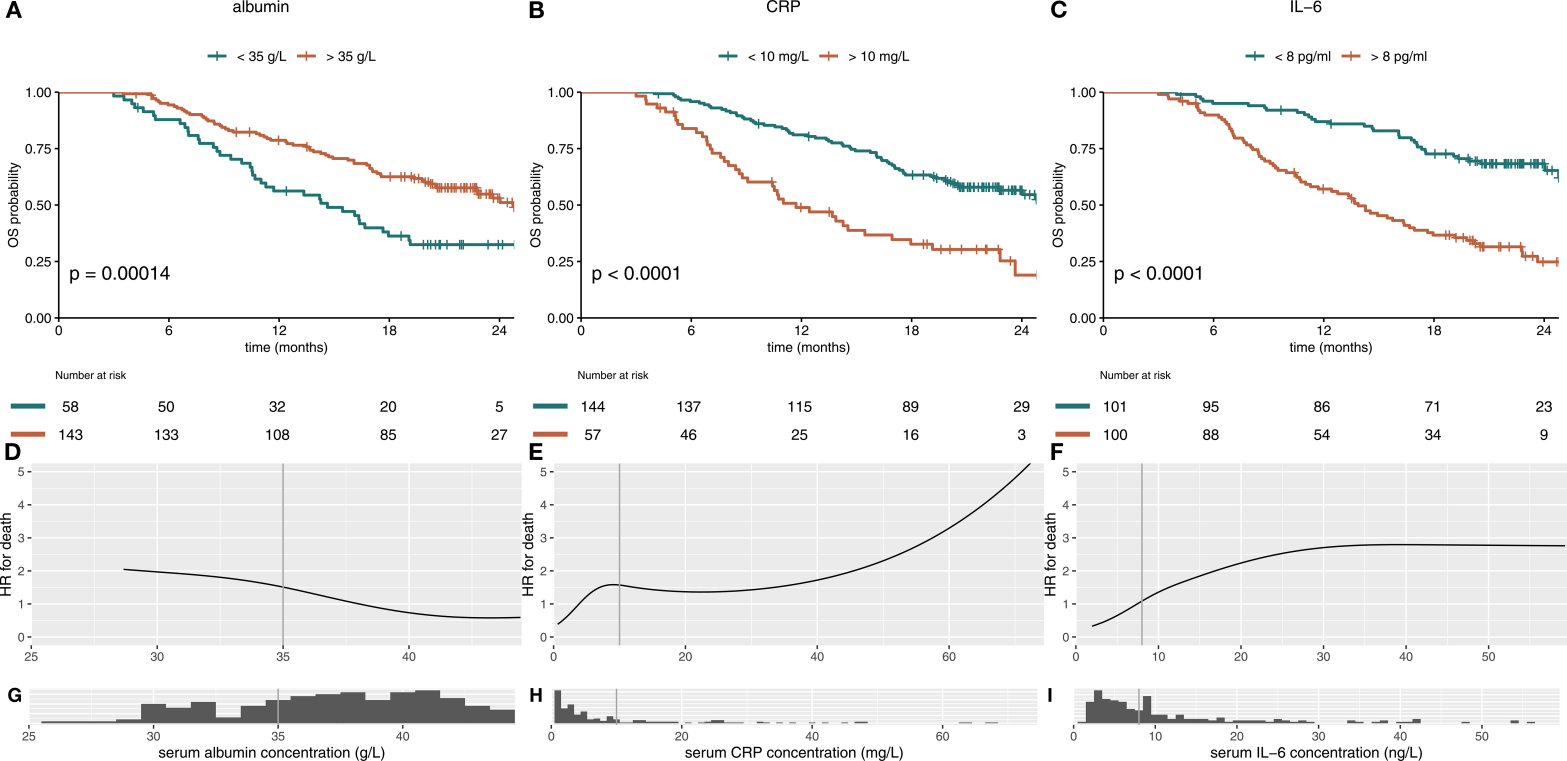
Albumin, CRP and IL-6 individually predict survival in immunotherapy-treated patients with HCC. Kaplan-Meier curves for serum albumin, CRP and IL-6 in immunotherapy-treated patients of the IMbrave150 trial at treatment cycle 4 are shown in **(A–C)**. **(A)** High albumin (>35 g/L) serum level and **(B)** low CRP (<10 mg/L) and **(C)** low IL-6 (<8 pg/ml) serum levels, calculated at treatment cycle 4, correlated statistically significant with better OS. The hazard-ratio (HR) for death was inversely corelated with albumin concentration with a linear shape **(D)**. For CRP and IL-6, higher serum levels were associated with impaired survival throughout the concentration spectrum **(E, F)**. Histograms show distribution of serum concentrations **(G–I)**. mGPS, modified Glasgow Prognostic Score; CRP, C-reactive protein; IL-6, interleukin 6; OS, overall survival.

Both parameters CRP and albumin at the beginning of cycle 4 correlated statistically significant with survival, when using the cutoffs relevant for the mGPS (p<0.001 **Figures 2A, B**). IL-6 as an alternative inflammation marker predicted survival better than CRP in the IMbrave150 immunotherapy cohort, when using a cutoff of 8 ng/L (**Figure 2C**). Analysis of the continuous variables revealed a strong negative correlation of albumin and HR for death (**Figure 2D**), while CRP and IL-6 show a positive correlation with HR. The HR for IL-6 shows a plateau at 30 ng/L (**Figures 2E, F**). However, most patients´ values were below the plateau concentration (**Figures 2G–I**). Changes in albumin, CRP and IL-6 on treatment are depicted in **Supplementary Figure S2**.

To improve the prognostic power of the on-treatment mGPS in the setting of HCC, we compared on-treatment mGPS with 2 other risk scores (ACR, AIR), which might potentially be superior in addressing the specific challenges of liver disease. ACR and AIR were divided into terciles with the following cutoffs: ACR low-risk 15.63 – 204.55, ACR intermediate-risk 4.53 – 15.62, ACR high-risk 0.18 – 4.52. For AIR cutoffs were 29.49 – 7.57 for low-risk, 3.24 – 7.56 for intermediate-risk and 0.06 – 3.23 for high-risk.

**Figure 3:**
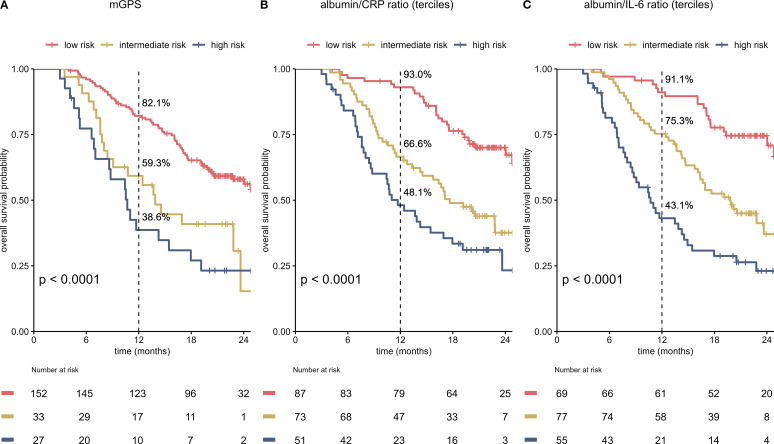
Albumin/CRP ratio (ACR) and albumin/IL-6 ratio (AIR) predict survival more accurately than the on-treatment mGPS **(A–C)**. Kaplan-Meier curves for the on-treatment mGPS **(A)**, ACR **(B)** and AIR **(C)** in patients in the immunotherapy arm of the IMbrave150 trial at cycle 4 are shown. All scores predict survival. Equal distribution of patients and better OS in low-risk patients result in an improved c-index for ACR and AIR when compared to the mGPS [c-index for ACR: 0.66 (95%CI 0.61 – 0.71), for AIR: 0.67 (0.62 – 0.72) for mGPS: 0.62 (0.57 – 0.66)]. mGPS, modified Glasgow Prognostic Score; CRP, C-reactive protein; IL-6, interleukin 6; OS, overall survival.

All scores, mGPS, albumin/CRP ratio (ACR) and albumin/IL-6 (AIR) ratio, correlated statistically significant with OS (**Figures 3A–C**). The mGPS classified most patients in the low-risk group, ACR and AIR were divided by terciles and therefore show an equal distribution of patients in the different risk categories. ACR and AIR exhibited superior prognostic information vs. mGPS in the high-risk group (HR for death 4.16 vs. 3.4, **Table 1**). ACR and AIR predicted outcomes more accurately than the mGPS (c-index for ACR: 0.66 (95%CI 0.61 – 0.71), for AIR: 0.67 (0.62 – 0.72) for mGPS: 0.62 (0.57 – 0.66)). Of note, when applying optimized cutoffs, defined by maximum likelihood-ratio test statistic based on a cox regression model, the prognostic value of ACR and AIR was further improved (**Supplementary Figure S3**). However, to avoid overfitting as an external validation dataset was not available, further analyses were performed using terciles. Prognostic performance of other, commonly used inflammatory scores are shown in **Supplementary Table S2**.

**Table 1:** Univariate cox proportional hazards regression analysis for OS and PFS stratified by mGPS, ACR and AIR at cycle 4.

Characteristic	OS				PFS			
	N	HR^1^	95% CI^1^	P-value	N	HR^1^	95% CI^1^	P-value
mGPS								
risk group								
low-risk	152	—	—		152	—	—	
intermediate-risk	33	2.31	1.39, 3.83	0.001	33	1.89	1.29, 2.76	0.001
high-risk	27	3.40	2.07, 5.59	<0.001	27	1.74	1.15, 2.64	0.009
ACR								
risk group								
low-risk	70	—	—		70	—	—	
intermediate-risk	70	2.16	1.26, 3.70	0.005	70	1.53	1.09, 2.16	0.014
high-risk	71	4.16	2.47, 7.02	<0.001	71	2.19	1.55, 3.09	<0.001
AIR								
risk group								
low-risk	67	—	—		67	—	—	
intermediate-risk	67	3.11	1.78, 5.45	<0.001	67	1.62	1.14, 2.31	0.007
high-risk	67	4.61	2.66, 8.00	<0.001	67	1.95	1.37, 2.77	<0.001

^1^HR, Hazard Ratio; CI, Confidence Interval.

**Figure 4:**
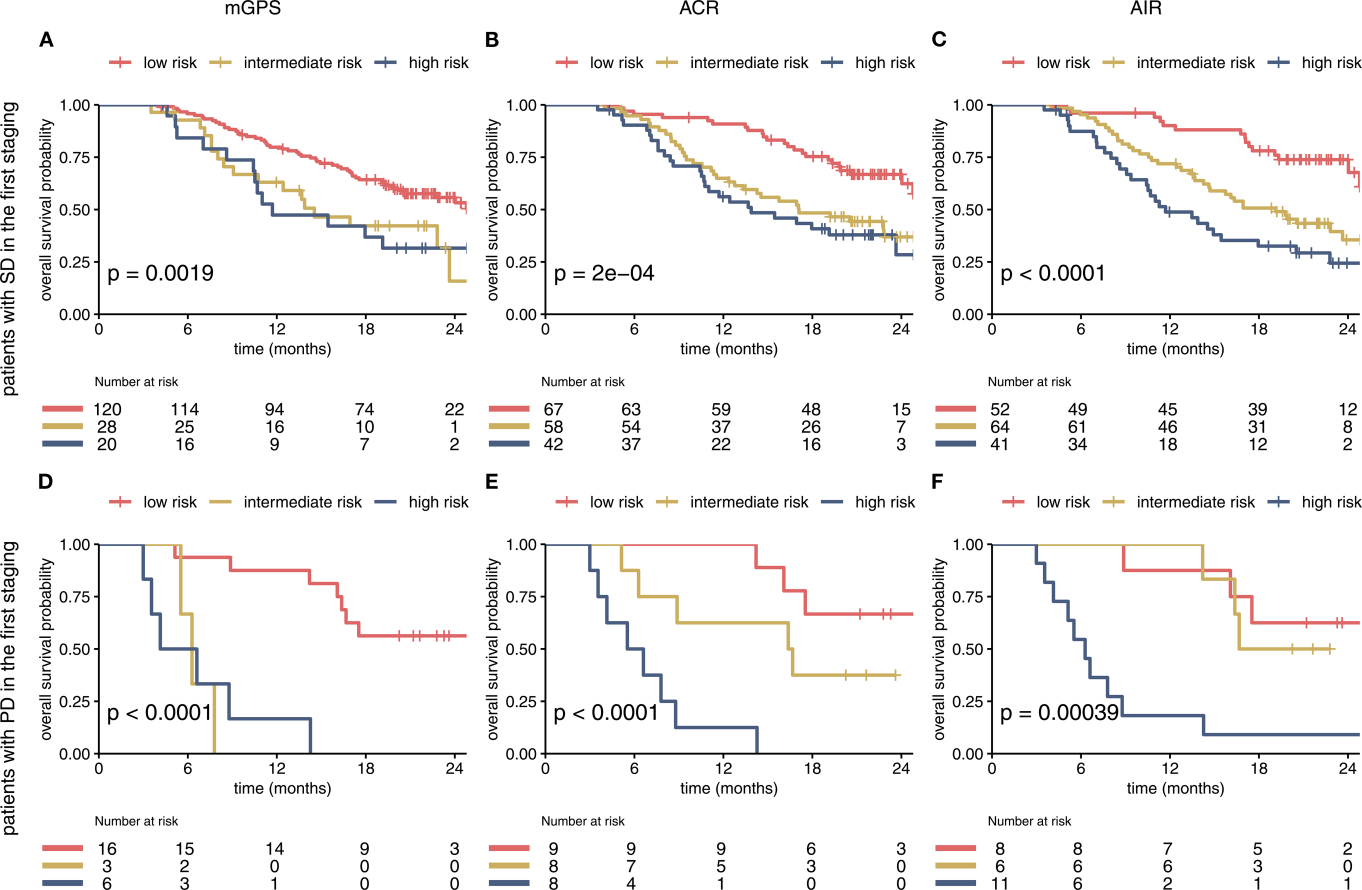
The mGPS, ACR and AIR predict survival within imaging-based subgroups **(A–F)**. Prognostic Information of on-treatment mGPS, ACR, AIR (at cycle 4) in the SD and PD subgroup of the IMbrave150 trial. All 3 scores at first staging have a strong prognostic value in the SD and PD subgroup. AIR and ACR identified a high-risk group of patients in the SD subgroup. mGPS, modified Glasgow Prognostic Score; ACR, albumin/CRP ratio; AIR, albumin/IL-6 ratio; SD, stable disease; PD, progressive disease.

To evaluate additional information of the different scores in conjunction with imaging, the on-treatment mGPS, ACR and AIR were compared within imaging-based subgroups. We found that each score can predict survival in patients with SD or PD in the first staging. The ACR and AIR were superior to the mGPS (**Figure 4**). The effect was retained in the multivariable analyses (**Table 2**).

**Table 2:** Multivariable Cox-regression for OS and PFS stratified by on-treatment scores and imaging.

Characteristic	OS												PFS											
	mGPS (OS)				ACR (OS)				AIR (OS)				mGPS (PFS)				ACR (PFS)				AIR (PFS)			
	N	HR^1^	95% CI^1^	P-value	N	HR^1^	95% CI^1^	P-value	N	HR^1^	95% CI^1^	P-value	N	HR^1^	95% CI^1^	P-value	N	HR^1^	95% CI^1^	P-value	N	HR^1^	95% CI^1^	P-value
Response in first staging																								
CR/PR	19	—	—		19	—	—		19	—	—	19	—	—		19	—	—		19	—	—		
SD	168	1.98	0.80, 4.92	0.14	167	1.71	0.68, 4.28	0.3	157	2.05	0.83, 5.08	0.12	168	1.42	0.88, 2.29	0.2	167	1.33	0.82, 2.16	0.2	157	1.33	0.82, 2.16	0.2
PD	25	3.61	1.32, 9.92	**0.013**	25	3.21	1.17, 8.79	**0.024**	25	3.22	1.17, 8.81	**0.023**	25	5.37	2.90, 9.92	**<0.001**	25	4.67	2.53, 8.64	**<0.001**	25	4.96	2.69, 9.16	**<0.001**
Score																								
low-risk	152	—	—		87	—	—		69	—	—		152	—	—		87	—	—		69	—	—	
intermediate-risk	33	2.43	1.45, 4.06	**<0.001**	73	2.58	1.59, 4.20	**<0.001**	77	2.71	1.58, 4.66	**<0.001**	33	2.01	1.37, 2.94	**<0.001**	73	1.47	1.06, 2.02	**0.020**	77	1.51	1.08, 2.11	**0.017**
high-risk	27	3.30	2.00, 5.46	**<0.001**	51	4.27	2.54, 7.17	**<0.001**	55	5.34	3.07, 9.31	**<0.001**	27	1.67	1.10, 2.54	**0.016**	51	2.02	1.41, 2.90	**<0.001**	55	2.21	1.53, 3.21	**<0.001**

^1^HR, Hazard Ratio; CI, Confidence Interval.Bold values indicate significant p-values (< 0.05).

## Discussion

In HCC, several baseline prognostic scores are used. The Barcelona Clinic Liver Cancer (BCLC) staging system guides therapeutic strategies by incorporating not only tumor-related factors but also liver function and ECOG performance status (15). Elevated serum alpha-fetoprotein (AFP) levels at diagnosis is known to be associated with poorer prognosis (16). Although biomarkers provide additional prognostic information, they do not generally guide clinical practice in advanced or metastatic settings (15).

In contrast to these baseline scores, on-treatment biomarkers can help to make clinical decisions during the course of therapy. This *post-hoc* investigation showed a good prognostic performance of on-treatment mGPS in HCC patients treated with atezolizumab and bevacizumab. Analyzing the individual impacts of CRP and albumin on the prognostic model revealed a statistically significant correlation with OS for both parameters. This is in line with the study of Kinoshita et al. showing good prognostic value of baseline mGPS in HCC (12). Our study is the first analysis of on-treatment mGPS in HCC, allowing for early detection of treatment resistance. Unlike prior studies that have predominantly focused on baseline or pre-treatment inflammatory markers, our study underscores the potential of the on-treatment mGPS as a dynamic, easily accessible, and cost-effective biomarker during treatment. Given its practicality, serial monitoring of mGPS during therapy could serve as a valuable tool for real-time clinical decision-making.

However, since the IMbrave150 trial only included patients with normal or mildly impaired liver function (Child-Pugh A), the value of those biomarkers may be limited in patients with more severely reduced liver synthesis. In HCC patients’ liver function is often impaired due to an associated underlying liver disease (e.g. hepatitis, nonalcoholic fatty liver disease) and CRP and albumin are primarily synthesized by hepatocytes (17). A previous study investigating CRP values in HCC patients in context of bacterial infection, showed lower increase of CRP in contrast to patients with normal liver function (18). Since the classification of patients into intermediate- and high-risk mGPS groups (mGPS>0) depends on an elevated CRP (>10 mg/L), it remains uncertain whether mGPS is reliable in HCC patients with severely reduced liver function (13). Yet, as patients with end-stage liver disease are not candidates for systemic immunotherapy, the study cohort of the IMbrave150 trial is representative for every-day clinical practice (4).

While the mGPS is a clinically practical and accessible prognostic tool, it may not fully reflect tumor-specific biological activity or liver functional reserve, being important factors for predicting outcomes in HCC. Furthermore, mGPS can be affected by non-cancer-related systemic inflammation or infections, which may compromise its specificity in the oncologic setting (12). To potentially improve the prognostic power of the on-treatment mGPS in the setting of HCC, we test the predictive value of IL-6 as an alternative inflammation marker. IL-6 was superior to CRP. It may overcome some of the limitations of CRP as a biomarker in HCC, since IL-6 is produced from extrahepatic cells, mainly immune cells. In line with this, the AIR predicted OS more accurately than the mGPS. However, measuring IL-6 is more costly and less broadly available compared to CRP (19). The comparison between the mGPS and the alternative score ACR demonstrated that the ACR exhibited superior efficacy in HCC patients. Since the mGPS only considers low albumin level in scoring when CRP level is high, we believe that the prognostic value of albumin is partly underrepresented in mGPS. In addition, ACR is divided into terciles as optimizing the cutoffs specifically for this dataset could lead to overfitting. However, the use of terciles yield arbitrary cutoffs which have not been validated on an independent dataset. Hence, this method may not generalize well beyond the current dataset. Alternatively, the mGPS scoring system, which has undergone broader validation, could offer more reliable results.

When sub-grouping the study population by imaging-based findings in the first staging, all 3 scores held prognostic power to predict OS within patients with PR and SD. This important additional information is of high clinical relevance to identify patients with higher risk profile. This could guide further management and imaging frequency in this vulnerable patient group showing a median survival of only 11 months (mGPS high-risk group).

When interpreting these results, it has to be considered that there was no separate validation dataset available for confirmation. It’s important to note that laboratory values were not accessible at the time of staging, which could impact the accuracy of the analysis.

As the gain in prognostic power by replacing CRP with IL-6 was minimal, and CRP is more widely available in clinical laboratories and more cost-effective, we consider the mGPS and ACR as the most clinically applicable score in patients with HCC. These data support the concept of integrating measurement of CRP and albumin levels in addition to radiologic staging to optimize treatment management in patients with HCC. Future perspectives should focus on integrating mGPS with other dynamic biomarkers, such as cytokine profiles or circulating tumor markers, as well as advanced radiological assessments. This multimodal approach could enhance the prognostic accuracy of mGPS, allowing for a more personalized risk stratification and treatment decision-making in HCC.

## Conclusion

On-treatment mGPS has been validated as a prognostic marker in the context of HCC treatment. While the CRP/Albumin ratio may offer potential advantages as a scoring system, it is not as widely adopted and validated as the mGPS. Replacing CRP with IL-6 have not yielded relevant clinical advantages, suggesting that CRP alone remains a reliable prognostic marker in HCC. Further trials are necessary to fully establish the role of on-treatment mGPS in guiding treatment decisions for HCC.

## Data Availability

Data from the phase III IMbrave150 trial supporting our findings (NCT03434379) were provided by Hoffmann—La Roche via Vivli by permission (Data Request ID 00008699) and are available upon request through Vivli, Inc (https://vivli.org/, vivli ID VIV00007372).
