# Supplementary material for: On-treatment modified Glasgow Prognostic Score (mGPS) in hepatocellular carcinoma treated with atezolizumab and bevacizumab provides prognostic information
Source: Front Immunol. 2025 Oct 9;16:1599143. doi: 10.3389/fimmu.2025.1599143 (PMC12546374; doi:10.3389/fimmu.2025.1599143)
Supplement: Supplementary file 1 [file DataSheet1.docx]

**Supplemental Information**

| **Characteristic** | **N = 212^1^** |
| --- | --- |
| Age | 65 (59, 71) |
| Sex |  |
| F | 37 (17%) |
| M | 175 (83%) |
| ECOG Performance Status(eCRF) |  |
| 0 | 141 (67%) |
| 1 | 71 (33%) |
| Child Pugh Score |  |
| 5 | 152 (72%) |
| 6 | 58 (28%) |
| Unknown | 2 |
| Etiology |  |
| Hepatitis B | 79 (37%) |
| Hepatitis C | 55 (26%) |
| Non-viral | 78 (37%) |
| Hist Grade of Cancer at Init Diagnosis |  |
| Moderately Differentiated | 45 (21%) |
| Poorly Differentiated | 20 (9.4%) |
| Unknown | 114 (53.9%) |
| Well Differentiated | 33 (16%) |
| De-Identified Region |  |
| Asia | 91 (43%) |
| Europe | 70 (33%) |
| North America | 47 (22%) |
| Oceania | 4 (1.9%) |
| AJCC TNM Stage at Diagnosis |  |
| STAGE IA | 15 (7.1%) |
| STAGE IB | 11 (5.2%) |
| STAGE II | 34 (16%) |
| STAGE IIIA | 26 (12%) |
| STAGE IIIB | 16 (7.5%) |
| STAGE IVA | 21 (9.9%) |
| STAGE IVB | 40 (19%) |
| UNKNOWN | 49 (23%) |
| BCLC Stage at Study Entry |  |
| STAGE A1 | 3 (1.4%) |
| STAGE A4 | 3 (1.4%) |
| STAGE B | 35 (17%) |
| STAGE C | 171 (81%) |
| Number of Metastatic Sites at Enrollment |  |
| 0 | 116 (55%) |
| 1 | 86 (41%) |
| 2 | 9 (4.2%) |
| 3 | 1 (0.5%) |
| PD-L1 Category 1 |  |
| Unknown | 111 (52%) |
| TC and IC < 1% | 35 (17%) |
| TC or IC >= 1% | 66 (31%) |
| Alcohol Use History |  |
| CURRENT | 39 (18%) |
| NEVER | 62 (29%) |
| PREVIOUS | 111 (52%) |
| Tobacco Use History |  |
| CURRENT | 41 (19%) |
| NEVER | 74 (35%) |
| PREVIOUS | 97 (46%) |
| ^1^ Median (IQR); n (%) | |

**Supplemental Table 1: Baseline characteristics of included patients**

| **score** | **c_index** | **lower_CI** | **upper_CI** |
| --- | --- | --- | --- |
| mGPS | 0.6154886 | 0.5688410 | 0.6621362 |
| AIR | 0.7000560 | 0.6460765 | 0.7540355 |
| ACR | 0.6879736 | 0.6366775 | 0.7392696 |
| GPS | 0.6329577 | 0.5836979 | 0.6822175 |
| NLR | 0.6168514 | 0.5429349 | 0.6907678 |
| PLR | 0.5712190 | 0.5032252 | 0.6392129 |
| LMR | 0.5853162 | 0.5108982 | 0.6597341 |

**Supplemental Table 2: Comparison of c-indices for commonly used inflammatory scores**


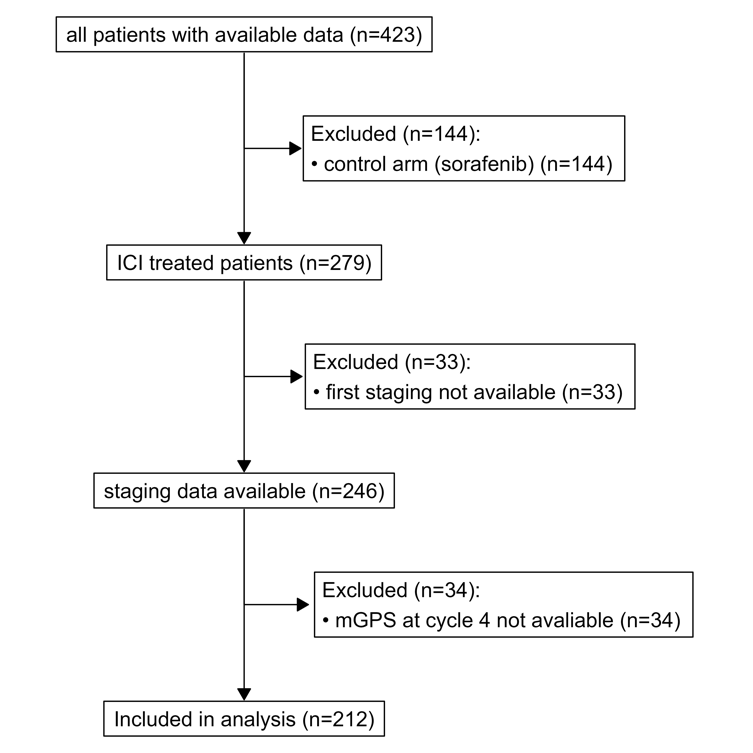


**Supplemental Figure 1: Consort diagram of patients included in the analysis.**

**
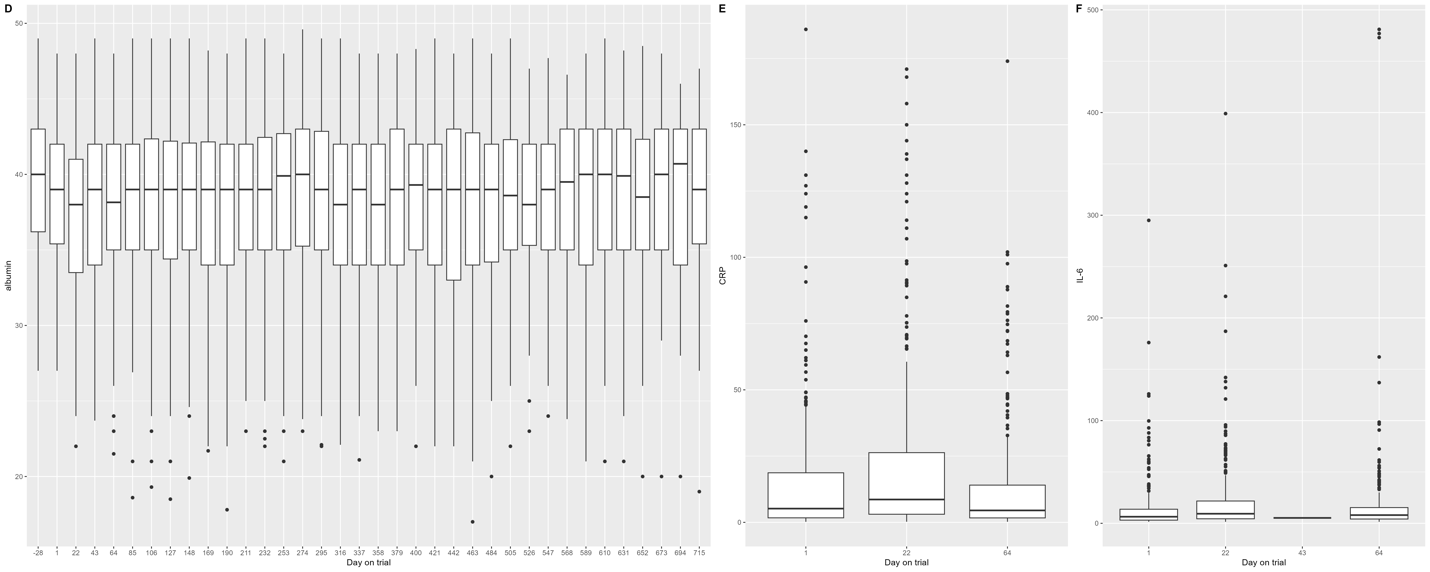
**

**Supplemental Figure 2: On-treatment changes of serum markers**

Individual (A-C) and aggregated (D-F) on-treatment changes of serum markers are shown. For albumin (A, D), all values within the first two years on treatment are shown. For CRP (B, E) and IL-6 (C, F), all available timepoints are shown.

**
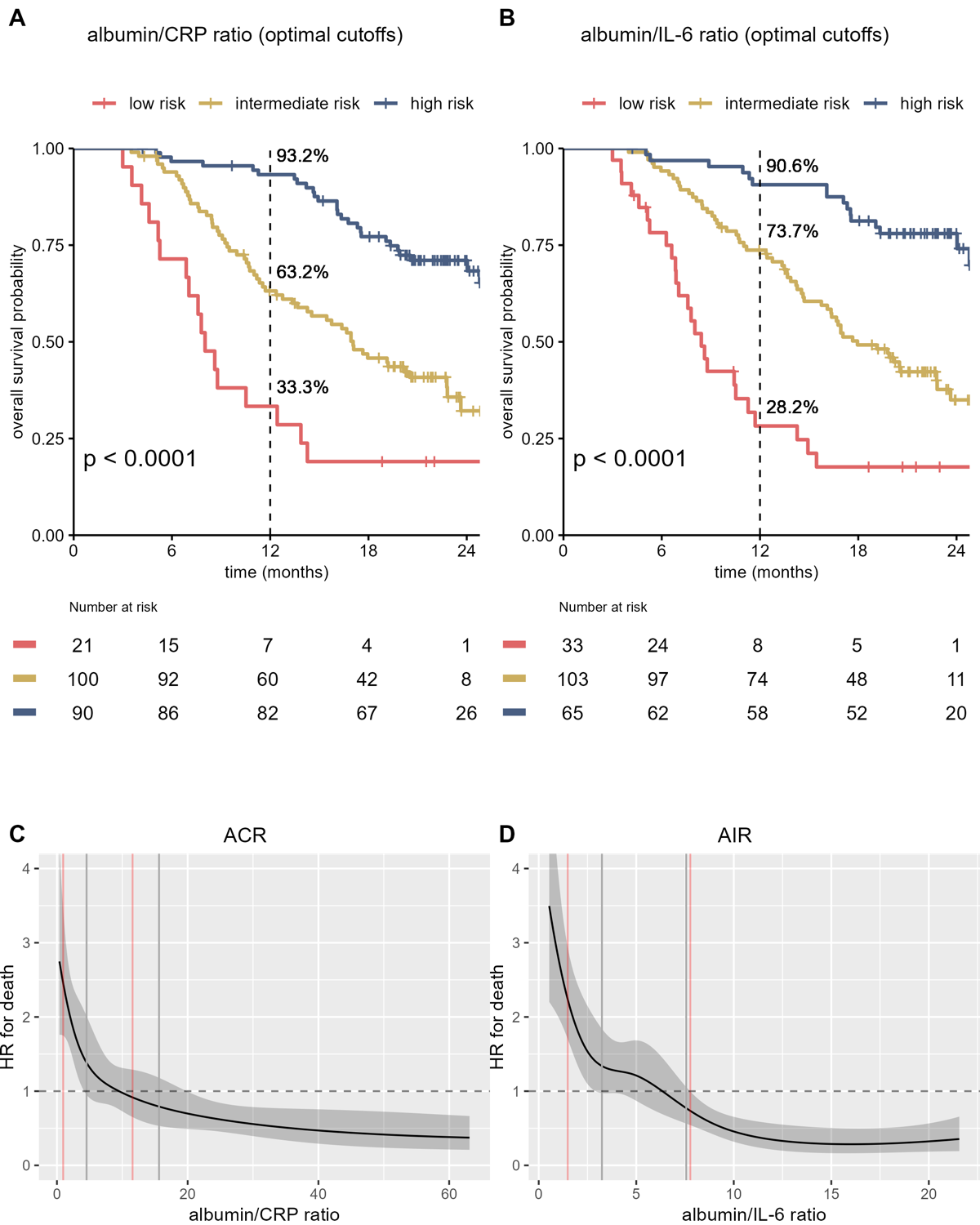
**

**Supplemental Figure 3: Optimal cutoffs for ACR and AIR**

Applying optimized cutoffs, defined by maximum likelihood-ratio test statistic based on a cox regression model, improves the prognostic value of ACR (A) and AIR (B). Both ACR (C) and AIR (D) have similar score-risk relationships as shown in the Hazard-Ratio plots. Cutoffs by terciles are shown in grey, optimal cutoffs in red.
